# Bread and Roses: A Gender Perspective on Environmental Justice and Public Health

**DOI:** 10.3390/ijerph13101005

**Published:** 2016-10-12

**Authors:** Karen Bell

**Affiliations:** School for Policy Studies, University of Bristol, Bristol BS8 1TZ, UK; karen.bell@bristol.ac.uk; Tel.: +44-117-331-0573

**Keywords:** equality, women, discrimination, environment, hazards, decision-making, feminism, ecology, capitalism, patriarchy

## Abstract

Gender continues to be a relatively marginal issue in environmental justice debates and yet it remains an important aspect of injustice. To help redress the balance, this article explores women’s experience of environmental justice through a review of the existing literature and the author’s prior qualitative research, as well as her experience of environmental activism. The analysis confirms that women tend to experience inequitable environmental burdens (distributional injustice); and are less likely than men to have control over environmental decisions (procedural injustice), both of which impact on their health (substantive injustice). It is argued that these injustices occur because women generally have lower incomes than men and are perceived as having less social status than their male counterparts as a result of entwined and entrenched capitalist and patriarchal processes. In the light of this analysis, it is proposed that environmental justice research, teaching, policy and practice should be made more gender aware and feminist orientated. This could support cross-cutting debates and activities in support of the radical social change necessary to bring about greater social and environmental justice more generally.

## 1. Introduction

Environmental justice is a contested concept but may be summarised as the multi-dimensional demand for, and/or achievement of, a healthy environment for all; equal access (across social groups) to environmental goods; equal protection from environmental harms; equal access to environmental information; and equal participation in environmental decision-making [1]. Of these various aspects, the majority of the environmental justice literature focuses on distributional justice, that is, distributional patterns among social categories [2]. This includes the distribution of environmental goods (e.g., energy, water and green space) as well as environmental burdens (e.g., air pollution, toxic chemicals and flood). However, it is increasingly being recognised that procedural justice, i.e., democratic and equitable control over environmental decision-making, is a necessary component of environmental justice [3]. Yet, even moving from distributional justice to include procedural justice would still only guarantee that we may participate in debates to secure a more equal allocation of environmental problems. Therefore, in addition to distributional and procedural justice, we also need substantive justice, i.e., the achievement of a healthy environment for all.

The social categories that have received the greatest attention with regard to environmental justice have historically been those linked to race and income, though in relatively recent years other categories have also been considered, including gender. Gender may be defined as “...a term used to emphasize that sex inequality is not caused by the anatomic and physiological differences that characterize men and women, but rather by the unequal and inequitable treatment socially accorded to them” [4] (p. 180). It is increasingly being seen as a diverse concept, with feminists emphasising the “intersectionality” of gender-based oppression, which “locates gender within contexts of race, ethnicity, class and citizenship among other socially constructed categories that interact to shape women’s lives and social identities” [5] (p. 382).

There has been substantial work on the gender aspects of environmentalism within the academic fields of eco-feminism, e.g., Nightingale 2006 [6], feminist environmentalism, e.g., Agarwal 1992 [7] and feminist political ecology, e.g., Rocheleau et al. 1996 [8]. These investigate women’s environments and women’s environmentalism but vary slightly in their emphases: Ecofeminism proposes that women’s domination under patriarchy is connected to the domination of nature; feminist environmentalism argues that women experience environmental degradation in gender-specific ways; and feminist political ecology focuses on gendered environmental politics and grassroots activism.

However, despite a long-standing line of enquiry into women and the environment which environmental justice researchers can build upon and borrow from, there have been relatively few publications on gender from within the environmental justice research field. The most notable examples, include Perkins 2015 [9], Perkins 2012 [10], Bell 2013 [11], Bell 2010 [12], Buckingham and Kulcur 2009 [13], Kurtz 2007 [14], Nightingale 2006 [6], Sze 2004 [15], Sze 2006 [16], Verchick 2004 [17], Warren 1991 [18], Platt 1997 [19], Kirk 1997 [20] and Krauss 1993 [21] among others mentioned in this paper. Buckingham and Kulcer [13] consider that this may be because women are not necessarily found in specific or segregated geographic locations. Their experience of environmental injustice may be at the scale of the household or body, rather than at wider scales. Hence, socio-spatial distributional analysis, which has been a dominant methodology in the environmental justice field, does not readily identify women’s environmental injustice. A gendered and/or feminist perspective is also generally absent from environmental justice policy, practice and activism. Gender issues are rarely articulated by those involved [22] and may even be suppressed [13]. Hence, this article contributes to an ongoing argument (e.g., [13]) about the need to further integrate a gendered perspective into environmental justice discourse, analysis and action.

A gender or feminist perspective is required in relation to environmental justice because women often live and work in circumstances that are not environmentally adequate and this has implications for their health (see “substantive justice” section of this paper). It is also the case that men can experience specific disproportionate environmental burdens, often as a result of their paid occupations [13]. However, here I focus on women since, as I will discuss, their environmental disadvantage and deprivation often goes unrecognised.

The article also highlights the improved public health outcomes that result from achieving environmental justice. It is increasingly being realised that health inequities between different social groups can be the result of environmental inequalities and injustices across social groups, e.g., [23]. Environmental justice discourse, when linked to debates about public health (i.e., preventing disease, prolonging life and promoting health), is ultimately radical because it de-individualises mainstream public health discourse (e.g., as presented in the mainstream media) with its focus on faulty genes and poor lifestyle choices as the causes of ill-health; and pharmaceuticals, operations and individual lifestyle change as the primary solutions. Instead, it encourages a focus on a holistic, preventative and contextualised view of health as resulting from, and/or facilitated by, a healthy environment. It does not necessarily dismiss the mainstream analysis of ill-health but can add to it, with environmental harms being seen as risk multipliers. Hence, a structural analysis based on an assessment of environmental injustices undermines the idea that gender differentials in health outcomes are natural and inevitable and implies that social change can improve women’s health outcomes.

In this article I draw together the relevant existing research, covering women’s lack of access to environmental resources; their inequitable burden of environmental harm; their specific “vulnerability” to environmental harm; their constrained influence over environmental decision making; and the health impacts of these environmental disadvantages. In addition, I draw on my own qualitative research over the last ten years (2006–2016), as well as personal experience as an environmental justice activist (for example, as convenor of the European Green Capital Inclusion Group (Bristol); as a “Diversity Champion” for the Green Party; and as the chairperson for an environment group on the Council estate where I live). I conclude with proposals regarding how women’s disadvantage with regard to environmental justice may be addressed. Whilst adding to the evidence base, the main contribution that this article makes is theoretical, arguing that women’s experience of environmental injustice is rooted in entwined processes of capitalism and patriarchy. This is a combined “socialist feminist” and “radical feminist” position (see Mackay for a thorough explanation of these terms) [24]. For this reason the paper is entitled “Bread and Roses” after the socialist feminist, Rose Schneiderman, who coined this term in her struggle to achieve healthier and safer workplace environments. As a member of the United States Women’s Trade Union League, she raised awareness of unsafe workplace conditions, following a factory fire which led to the death of 146 garment workers, 123 of which were women.

## 2. Method

A comprehensive search strategy for the literature review was developed to locate all the relevant published evidence to date. This included: “grey” literature, government reports; theses; and conference reports. The evidence was identified though social science database searches and citation tracking, using search terms selected to ensure maximum sensitivity and specificity. This was followed by quality appraisal of the literature generated. The relevant data was then extracted, synthesised and thematically coded. Subsequently, I looked at my own research on environmental justice over the last ten years (2006–2016), reanalysing some of the qualitative elements from a gendered perspective including that published in Bell 2008 [25]; Bell and Sweeting 2014 [26]; and Bell 2014 [1].

I also included knowledge gained in relation to my environmental justice activism over the same period, which had been recorded in notes, emails, letters, webpages and blogs. The latter included my activities and involvement in meetings, events and activities related to political parties, local government, community events, campaigns and activist groups. The identities of individuals referred to in these contexts will be anonymised throughout to avoid any unwanted publicity for those mentioned. Although all the meetings and communications cited here were public and not confidential, it was not always possible to gain full informed consent from participants in these encounters. Therefore, it seems reasonable and ethical to protect them from any harm or unwanted attention that could arise from repeating their words outside of the fora where they were originally articulated.

I used the technique of “framework analysis” to reanalyse the qualitative research data and the activist material, as described by Richie and Lewis [27]. This technique allows themes to emerge from the research questions, the literature, and the research participants. Hence the study used a triangulated combination of established social research methods.

Finally, in the feminist tradition, I would like to situate myself in the study in terms of my own background. I am a middle-aged woman from a manual working class background and of Romany Gypsy heritage. I have lived most of my life on Council estates, that is, areas of predominantly public or social housing built by local authorities to house mostly working class people. My work experience has been diverse and, prior to academic work, included factory production using toxic chemicals and environmental community development in working class areas. I have been an environmental and social justice activist most of my adult life.

## 3. Evidence

This section presents the evidence for the argument that a gender dimension to environmental justice needs to be more thoroughly explored and articulated in political arenas as well as the evidence regarding women’s experience of distributional, procedural and substantive environmental injustice. Each aspect is discussed separately but it is important to note that they interact and can, therefore, reinforce as well as offset each other.

### 3.1. Linking Gender and Environmental Justice

Women have traditionally been the leaders and activists in the environmental justice movement [28]. It is speculated that this is because men have more of a vested interest in the economic and political institutions responsible for environmental harms [17]. Those who are not well represented in the current structures of power find it easier to see when the system is not working for them [21] and are, therefore, more likely to challenge it. However, women involved in the environmental justice movement do not generally seem to assert a gender dimension to the topic or to articulate the gender issues involved [22]. In general, a lack of gender and equalities awareness prevails in environmental institutions and environmental activist groups. For example, Buckingham and Kulcur assert: “...within the environmental movement gender difference has been suppressed in the name of unity and solidarity with humanity in general” [13]. Even so, this is not to say that women in environmental and environmental justice organisations are not feminists or that they do not have an understanding of gender issues. There is some evidence to suggest that they do. For example, Berlia [29] points to the way that women in environmental justice organizations employ feminist tactics of collaboration and coproduction of knowledge, developing their own shared knowledge base which can counter that of the “experts”. However, it appears that, in general, female environmentalists are not (yet) strongly considering the gender aspects of their work. Similarly, women’s liberation organisations and gender academics have not tended to take a strong interest in environmental issues. This may be because environmentalists, in general, have not traditionally taken up the issue of feminism and women’s rights [30].

My personal experience as an environmental activist supports these findings, as illustrated in the following example. When Bristol, my home town, received the European Green Capital award for 2015, an “Inclusion Group” was set up, ostensibly to ensure that the programme of activities would represent the interests, and cater to the needs, of diverse social groups. However, the initial Inclusion Group meeting attracted mainly representatives of local environmental NGOs, as well as some city councillors, council officers and a few community group leaders. There were very few “equalities” leaders—i.e., those representing groups that may experience less favourable treatment on the basis of their gender, race, age, sexual orientation, origin, class, income, language, religion, convictions, opinions, health or impairment. The attendees expressed their purpose for being there in terms of “getting lots of people interested in Green Capital” (comment from first Green Capital Inclusion Group attendee, 2014). There appeared to be virtually no understanding of any equalities aspect to “inclusion” or “Green” on the part of the majority of the group, including in relation to gender. When I endeavoured, by making suggestions at the first three meetings, to ensure that Bristol’s equalities groups were involved (e.g., Bristol Women’s Voice, the main local forum for women’s interests), there was resistance from the majority of these environmentally motivated attendees. I was told initially that it was “not necessary” and later that it was “too late” (2nd meeting of Green Capital Inclusion Group) to involve these groups. Throughout the meetings, when I continued to speak on the opportunities implicit in Green Capital for tackling the environmental injustices faced by equalities groups, including for women, dismissive comments were made such as that we should be “celebrating the resilience” of these groups, rather than pointing out injustices (3rd Green Capital Inclusion Group). When I was finally permitted to approach Bristol Women’s Voice, they responded very positively and immediately sent a representative to the Inclusion Group meetings. However, the representative articulated very few gender issues in relation to Green Capital (mostly in relation to the time of the meetings) and raised none of the wider environmental and environmental justice issues that pertain to women, such as those that I outline in this article. This observation of there being little understanding of equalities among environmentalists and limited understanding of environmentalism among equalities groups echoed that of numerous other occasions when I worked with and within environmental and social justice groups.

Hence, a review of the literature and personal experience suggests that a gender dimension to environmental justice is currently not well articulated in policy and activist circles and is marginal in the environmental justice literature. The reason that this is important is because women do not currently experience environmental justice as the next sections discusses.

### 3.2. Distributional Environmental Justice

There is some evidence from the environmental justice, geography and development fields that there are gender disparities in terms of environmental burdens and access to environmental goods. Female headed households are more likely to be found in polluted and environmentally degraded areas [31]. They are also more likely to be subject to indoor air pollution as a result of their cooking responsibilities and the use of solid fuel cookers [32,33]. In addition, women are less likely to be able to afford to access adequate food; energy for heating and cooking; parks and green spaces and other environmental resources). For example, they are more likely to experience fuel poverty because of their lower incomes and increased likelihood of living in draughty, poorly insulated or damp accommodation [31]. Women are also more likely to make food sacrifices for other family members when there are food shortages in a household [34]. Furthermore, they are more likely to be impacted by climate change, since it is a risk multiplier, impacting on populations that are already disadvantaged [35,36]. When environmental disasters such as earthquakes and hurricanes occur, evidence suggests that women are at greater risk than men with higher mortality rates [37]. There is also evidence that male violence against women increases in the wake of “natural” disasters as a result of women’s oppression more generally and their lack of inclusion in disaster responses [38]. In general, women are less likely to have the resources to adapt to or avoid environmental problems [35]. For example, they may not be able to afford to retrofit their homes to reduce the impact of heatwaves.

Women may also have different environmental needs to men. For example, internationally, men use cars more than women [39,40]. Where there is only one car in a household, it will usually be used by the adult male [39]. Hence, women are more likely than men to use public transport [39,40] and they will prefer it to be supported by good security, such as adequate staffing, because they may restrict their travel if they feel they might encounter a dangerous situation [39]. Therefore, improved and well-staffed public transport will be of most benefit to women, whilst targeting resources at facilities for car-drivers will tend to be of most benefit to men.

Several studies also highlight the burden placed on women to carry out environmental protection activities, such as recycling or energy efficiency [31,41]. In the developed world, women must decide if they can afford the time, financial cost and physical energy to buy organic food, compost green waste, recycle, cook fresh food, and use reusable nappies, menstrual products and other items when there are disposable alternatives available. Women’s traditional role in household management presents opportunities for them to influence lifestyle activities, such as food choices and levels of recycling. However, only focusing at this level strengthens gender stereotypes and may, in some cases, increase their domestic workload [13]. As Buckingham and Kulcur [13] point out, until there is a fair and equitable division of labour in the home, the burden of household level environmental activities will fall to women. Recent studies show that women also tend to experience “time poverty” as a result of, for example, caring responsibilities, or long hours of work [42]. Therefore, these extra environmental responsibilities may place a strain on women. Moreover, some of these environmental activities are very individualised answers to environmental problems. For example, recycling of waste allows corporations and governments to put some of the costs of waste management onto the female public and shields them from demands to reduce or reuse packaging or change environmentally damaging production such as planned obsolescence.

Hence, the literature evidences the disparate environmental burdens borne by women. My own research activity supports this. For example, in 2007 and 2013, I interviewed a group of women who had set up an environment group on a Bristol council estate, one of the 10% most deprived in the country. It was evident that the environmental services were not adequate or appropriate for them. In relation to their large-item and green waste removal, which householders have to pay for in Bristol, a female interviewee said:

*The green stuff from the garden, garden waste, they don’t come and collect it. You have to pay and I can’t afford it...On a Sunday night it’s like Beirut with all the garden waste being burnt so it is a big issue, you know, how to get rid of it... There’s fires all through the summer…one day they had to get the fire brigade out. People are burning the waste because they can’t afford to get it collected*.

[26]

The respondents also alluded to the burden of environmental activities when combined with a shortage of time. For example, one resident asserted:

*It’s putting everything on the individual. I mean, why should we be messing around with waste? The Council don’t have to do so much now as we have to spend time sorting it and it’s like with the electric, before they used to come and read the meters, they employed people to do that. Now they’ve laid them all off and we have to do it ourselves*.

[26]

Hence, we see that women experience particular environmental burdens, resource inequities and responsibility for environmental protection. It should also be noted, though there is not the space to explore this here, the degree to which women experience distributional environmental injustice, and the form that it takes may be compounded or shaped by intersectional factors, i.e., their membership of other disadvantaged and marginalized groups, including ethnic minority, disabled, young, old and LGBT (Lesbian, Gay, Bisexual and Transgender) groups.

### 3.3. Procedural Environmental Justice

As discussed earlier, women make up the majority of those involved in the environmental justice movement and at the grassroots, community and membership level of environmental action groups. However, in the more formal, well-financed and mainstream sectors of environmentalism, environmental decision-making is generally dominated by men. In environmental NGOs boards and leadership and management roles in the resources, energy, and planning sectors, as well as in international climate change negotiations, women are vastly under-represented [43]. For example, a 2006 survey of 17 environment ministries in developing countries showed that women made up 41 percent of the staff but only 27 percent of the management [44].

Governmental and supra-governmental organisations are increasingly drawing environmental non-governmental organisations (ENGOs) into decision-making and policy-making. However, they tend to depend on leaders from the ENGOs that are best resourced. This tends to skew participation towards males who are more likely to take up the senior roles in the most prominent institutions. Smaller women’s organisations are less likely to have the resources to participate in activities where there is no financial recompense [13]. It is also widely acknowledged that women have limited availability for meetings because of the demands of the “double day” of paid and domestic work [45,46]. In addition, though there are some women’s environmental organisations, they do not necessarily take a feminist perspective in the sense of addressing patriarchal structures in environmental decision-making and campaigning fora. For example, the Bristol-based, “Women in Sustainability” network, whilst recognising that men dominate the field, mainly focus on developing the skills and networks of women, rather than challenging social structures [47].

Yet, even where woman are well represented in a decision making forum in terms of numbers and where they can articulate a feminist or equalities perspective, they are often silenced or ignored. A number of writers have discussed how this happens in society, more generally. For example, Ballard-Reisch [48] describes how those who are members of subordinate groups don’t speak up on the issues they face. Houston and Kramarae [49] posit that women are silenced in many ways, through, for example, ridiculing and trivializing their opinions, ideas, and concerns. Male gatekeepers mute non-dominant groups, especially women, in a number of areas of public life.

The notion that women are not being taken seriously by men in environmental discussion meetings is a common theme in the literature on gender and environmental justice. For example, a female US environmental justice leader who participated in Snyder’s [22] study commented:

*Most of the agencies... local governments, state, and federal governments are all run by men... And they tend to look down on women or minimize their information or their knowledge or their voice*.

(Lopez 12/02/2011 in [22])

In a similar vein, the women that took part in our research on environmental justice on a Bristol council estate spoke about how their input to Neighbourhood Renewal decision making meetings was disregarded and manipulated; and how disempowering this was for them:

*They seem to walk all over us or send us people who don’t care who aren’t from the area. They go back and say what we want and then it just gets chucked in the bin*.

[26]

*There’s a lot of muddying the waters that goes on so you can’t really follow what’s happening. They don’t follow the processes they are supposed to or reply to things. You just end up getting confused and give up*.

[11]

*I’ve noticed a distinct increase in bullying, instead of calm discussion* [in the Council meetings I go to]. *You end up feeling hurt and disgusted and demoralised*.

[26]

My experience with the Green Capital Inclusion Group is also illustrative of the themes found in my research and in the literature on gender-based environmental injustice. For example, men dominated the group, even though they did not make up the majority of those present. Despite most attendees being female, in general, three particular middle class males dominated the conversations each month. They spoke in relation to almost every agenda item, even though the agenda was always full and the meetings large. On one occasion, when I was asked to chair the meeting, I moved on to the next agenda item even though one of these males had raised his hand to speak about the current item. On this occasion we were running late so to move on seemed a legitimate decision. However, he immediately interrupted me, complained about unfair treatment in not having the space to speak whenever he wanted and stopped me moving on, making his point despite my decision.

My experience as a member and activist in the Green Party also illustrates these dynamics. As a former equalities trainer and advisor in a number of community development roles, I was keen to use my knowledge and skills to help create a more diverse party. For several years I tried to inject some equalities awareness into my local Green Party group. In 2011, the Green Party finally created a “Diversity Champion” network and I was selected to be one of the Champions. We were not issued with job descriptions, but I assumed my job was to ensure that more people from equalities groups were able to join and influence the party and its policies. However, despite being given this role, none of my advice was heeded. The extent of my frustration is illustrated in the email I sent on 7 July 2012 to the Green Party’s National Diversity Champion Coordinator:

Dear Gayle, I am struggling with my “diversity champion” role as I don’t seem to get listened to. I think the local party think I am going to bring in all the working class and black people and they can just carry on doing what they are doing… [even though some of what they do is]…creating the barriers to people getting involved… We need support as many of us are from oppressed groups and cannot be expected to take on class and race prejudice and ignorance etc single handedly, as the dominant groups are in the habit of ignoring us.

No adequate support was forthcoming so, shortly after sending this email, I resigned from the post in frustration and later I resigned from the party.

As chair of the Lockleaze Environment Group, from 2007–2010, I also witnessed women’s views being ignored. For example, female members of our group participated in a three year consultation process regarding a neighbourhood plan for Lockleaze (the Lockleaze Vision). We raised a number of issues, some of which are relevant to gender, such as the need to preserve the small green spaces near our houses, since most of us were not very mobile and valued local greens for our children’s play and our own wellbeing. Many of these green small spaces were, at that time, under threat because house prices were soaring and the Council wished to sell off the land to private developers. They also wished to build houses next to the High Voltage Transmission pylons that run through the estate and we argued against this because the Electro-Magnetic Frequencies produced by these facilities have been found to cause miscarriages and depression, as well as cancer and leukemia. The male Lockleaze Programme Manager consistently dismissed the issues we raised during the process and frequently failed to record them. At the end of the three year period, he told us that none of our input would be integrated into the planning document and that it would appear only as an appendix item [50]. Hence, in Appendix B of this document is a statement from me which includes the following:

*No one I speak to in Lockleaze Environment Group or my other neighbours want or need any of this and it is frustrating to keep saying this without it ever being noted. We need more and better services and facilities, not more property development, roads and car parks...We want our voice to make a difference. I have been very actively involved, attending most of the meetings... I have made all these comments on many occasions at the meetings but they seem to have somehow disappeared or been distorted in the final document. Lockleaze Environment Group has not been mentioned once in this document*.

[50]

In the examples taken from my personal experience and research, it was by no means clear that it was gender that prevented myself and the other women having influence, rather than class (we are all from working class backgrounds and live on Council estates) or ethnicity (we are from a range of ethnic backgrounds) or some aspect of our personalities, or the fact that we were challenging the status quo or threatening business and financial interests. However, in the context of all that is known about women’s voices being ignored, our gender should be considered as a possible factor that led to our views being discounted by the men controlling these fora.

Hence, women appear to experience procedural environmental injustice in terms of being under-represented in influential roles and silenced and ignored in decision-making fora. This, in turn, impacts on their opportunities to access environmental goods and avoid environmental burdens, relative to men, and, thereby, their health and wellbeing, the topic of the next section.

### 3.4. Substantive Environmental Justice

Substantive environmental justice is about all social groups living in a healthy environment. Hence, achieving substantive environmental justice should lead to better and more equal health outcomes. Gender inequalities in health have been a major area of research interest since the early 1970s. It is generally considered that, while women have lower mortality rates than men, they also experience greater morbidity [51]. This gives rise to the somewhat contentious notion that “men die quicker but women are sicker” [52], In addition to the overall mortality and morbidity rates, certain health issues can be more commonly associated with one gender. For example, dementia, depression and arthritis are thought to be more common in women, while lung cancer, cardiovascular disease and suicide are considered to be more prevalent in men [53]. Feminist research on gender differences in health has distinguished between biological and social issues, making it clear that gender inequalities in health were mainly socially produced, rather than biologically given. As such, they could be addressed through policy and ameliorated or eradicated, once the social determinants of ill-health were understood. 

However, mainstream health discourse (e.g., as found in the mainstream media), even where it recognises the socially produced determinants of health, tends to downplay the role of environmental factors. In recent years there has been a surge in the morbidity and mortality rates of non-communicable diseases—cardiovascular diseases, diabetes, cancer and chronic respiratory diseases [54,55,56,57]. For example, cancer incidence rates which, worldwide, have continued to increase to the extent that there is now an 18.6 percent chance of developing the disease before the age of 75 (29.9 percent in US, 26.3 percent in UK) [58]. Yet this surge is rarely publicly explained in terms of environmental factors. We are repeatedly told that the reasons for the global increase in all these diseases are our lifestyles, our genes, our increasingly long lives and more intensive diagnosing, e.g., [56,59].

This contradicts the numerous studies which have linked these non-communicable disease epidemics to toxic substances in the water we drink, the food we eat, the products we use, and the air we breathe. For example, the following illnesses have been linked to air pollution, alone: heart disease [57]; cancer [60]; strokes [61]; pneumonia [62]; respiratory illness [63]; adverse birth outcomes [63]; impairment of cognitive development [64]; diabetes [65]; depression and suicide [66]; impeded brain development in children [67]. The World Health Organization (WHO) suggests that 23 percent of global deaths are attributable to environmental factors [68]. The International Labour Organisation reports that, globally, a worker dies from a work-related disease or accident every 15 s, amounting to more than 2.3 million deaths a year [69]. These studies may even underestimate the burden of disease and death attributable to environmental causes, as only a fraction of the potential risks have been adequately investigated.

We have a situation where these non-communicable diseases are devastating almost all our lives, if not our own bodies, much as the AIDs epidemic of the 1980s shattered whole communities, but because the causes of these epidemics are presented in individualised and fatalistic terms, public anger is avoided. There has been little encouragement to link these non-communicable disease outcomes to the demise of our environments, which might well provoke such anger. Though this denial of the environmental determinants of ill-health applies as much to women’s as men’s health, it important to raise the issue as a prelude to discussing women’s health so that there is an understanding of the importance of creating healthy environments.

As women’s health is such a large topic, I wish to focus here, by way of example, on breast cancer, since it is the leading cause of deaths from cancer in women, both in low and high income countries, contributing to over 508,000 women’s deaths in 2011 [55,70].

There is some evidence of a strong environmental component to breast cancer [71]. For example, research undertaken by the Alliance for Cancer Prevention and the UK Working Group on the Primary Prevention of Breast Cancer indicates that lifelong, low-level everyday exposure to toxic substances and hormone-disruptors—from pesticide residues in food to chemicals in cleaning and body products and in the workplace—are linked to the ever-rising rates of breast cancer [72]. Though research on women’s occupational health is generally lacking, some research has specifically linked breast cancer to the work environment. Though there are numerous different work environments involved, for reasons of space, I will focus here on the plastics industry as it has a very high concentration of women workers [73].

Plastics processing exposes workers to a multitude of toxic chemicals, including styrene, acrylonitrile, vinyl chloride, phthalates, bisphenol-A (BPA), brominated flame retardants, heavy metals and solvents. All of these substances are either known to, or suspected to, cause cancer in humans. Epidemiologic evidence indicates that women in the plastics industry are experiencing breast cancer at elevated rates as a result of workplace exposure to these substances. For example, a 2012 Canadian research review found an increased risk of breast cancer among women working in this industry [73]. Another study showed that incidences for premenopausal women who work in food canning, where they may be exposed to BPA from the tin linings, was more than five times that of the general female population [74]. Yet another study [75] of women working as plastics processing machine operators found a doubling of breast cancer risk. Labreche et al. [76] and Shaham et al. [77] also reported an excess risk of breast cancer with occupational exposures to plastics-based synthetic textile fibres.

Hence, we can see one area of occupational activity and its impact on one particular disease that overwhelmingly impacts on women. This is but one illustration of how women’s health is being undermined by an inadequate environment. It also, by implication, indicates that when women experience distributional and procedural environmental injustice, this will have consequences for their health, both relatively and absolutely. The next section discusses the probable reasons for these distributional, procedural and substantive environmental injustices.

## 4. Discussion

### 4.1. Explanations for Gender-Based Environmental Injustices

Environmental injustice is usually framed as being caused by one or a number of a combination of the following factors: discrimination, power inequities, industrialisation, market dynamics, lifestyles, discourses and capitalism [1]. With regard to environmental injustice in relation to gender, some also argue that there are biological reasons for gender differentials. For example, with regard to breast cancer, there may be hormonal differences and different sensitivities due to differences in detoxifying activity. Women usually have a higher percentage of body fat than men, which has been associated with storage of lipophilic chemicals [23]. This has led some to describe women in terms of being more vulnerable than men. However, the notion of discussing women and environmental justice in terms of “vulnerability” plays into stereotypes of women as weak and in need of protection. Men will have different “vulnerabilities”, though men are almost never referred to as being more vulnerable than women. Buckingham and Kulcur [13] also note the tendency to “other” women, i.e., to construct females as “different” to a male norm, much as disabled people are constructed as aberrations to “normal” [78].

I would argue that economic and social reasons generally best explain what might be perceived as women’s relative “vulnerability”. For example, where women are more likely than men to die in “natural” disasters, this may be interpreted as biological “vulnerability”. However, evidence suggests that this largely occurs as a result of socially determined gender roles and low income. Because women are generally poorer than men, they are more likely to live in unsafe housing and in risk-prone areas [36,37]. Their gender role also means that they are more likely to be with children and others they are caring for and less likely to have access to private transport with which they could escape [79,80].

I would further argue that underlying all the factors posited as explaining environmental injustice in respect to gender, there are two main issues—low income and low status.

Many of the inequitable burdens experienced by women occur because of the “feminisation of poverty”—i.e., because women tend to be less wealthy than men [35]. This occurs on account of their being more likely to be single parents [81] and carers. In addition, women are often low paid employees [35,80] since discrimination in the workplace impacts on their employment promotion prospects. All these factors result in women’s reduced income, ability to save and wealth. Gender norms, constructing women as carers, means that they will take on the majority of the responsibility for unpaid care of children and ill or disabled adults. Caring responsibilities can impact on labour participation rates and, thereby, income and wealth. This, in turn, impacts on their ability to access environmental justice because low income groups are more likely to experience a whole series of environmental problems. Poverty reduces the ability to choose healthy working and living conditions, as there is little or no scope for rejecting the little that is available and affordable. In the United Kingdom it has been found that poorer people experience more environmental burdens and less environmental goods as evident, for example, in studies on environmental injustice in relation to air pollution [82], transport [83], proximity to landfill sites [84], flood risk [85], food poverty [86], fuel poverty [87], access to green space [88], and multiple inequitable burdens [89].

Poverty interacts with status to set the pattern for gender inequality. Hence, for example, though food insecurity is mainly a result of poverty [36], women’s socialization to put others first means that they carry the burden of this insecurity. Additionally, as a non-dominant group, women’s voices often go unheard when they complain about the environmental injustices that are inflicted upon them and when they try to engage with the structures that are responsible for the injustices as discussed earlier.

Race, class, disability, LGBT identity, age and other intersectional considerations will also shape environmental justice outcomes for women. These factors often compound the environmental injustices that some women experience since, in many instances, they go hand in hand with poverty and unequal status. However, because there tend to be more or less equal numbers of men and women in each of these categories (with the exception of older age), they are not discussed here as causes of gender-based environmental injustice. Their role is to shape, and in many cases, compound such injustice. Even so, it is important to note that using an environmental justice framework that focuses solely on any single issue, will not be sufficient to capture the form and extent of environmental injustice that most people experience.

Hence, though other factors are influential, this analysis indicates that poverty and unequal status are the primary factors driving gender-based environmental injustice. The causes of this poverty and inequality, I would argue, are the processes of capitalism and patriarchy.

The argument that capitalism is a cause of environmental injustice is based on the claim that it, not only causes environmental damage, but also that it tends to polarise income and wealth inequalities, creates unemployment and underemployment and is prone to periodic crisis which tend to be borne by the worst off. The class divisions that occur in a capitalist society favour some lives and marginalise others and benefits and costs are distributed according to purchasing power. There is no rational way to prioritize under a capitalist system, in which the market decides how commodities are allocated. Poor people do not obtain necessary goods because access to commodities is determined, not by desire or need, but by having sufficient money or credit to make purchases. At the same time, wealth is taken from the working classes and siphoned off to elites, enabling vast concentrations of wealth. A system that produces inequality and depresses workers’ wages ensures that many will not have access to even the basic necessities.

Capitalism has led to unprecedented levels of income inequality and, when combined with patriarchy, this means that women will be disadvantaged and, in many cases, poor. Under capitalist and patriarchal systems, gender is a basic organizing principle for allocating duties, rights, rewards, and power. Capitalism is organised around the goal of accumulation, not the health and material needs of people.

In addition, capitalism restricts the possibilities for procedural environmental justice [1]. A market economy requires profit and growth so that these priorities come to dominate decision making, at the expense of ecological and social concerns. Democracy under capitalism is limited because there is a ruling class who work to control society in their own interests. The capitalist class has the most resources to spend on behalf of its interests and are able to subvert local and national democracy through funding anti-environmental movements and election candidates and forming powerful lobbies to shape government policies to protect their economic interests [90,91]. For example, Monbiot documents numerous examples where big business has over-ruled parliamentary democracy in the UK and public provision “...*has been deliberately tailored to meet corporate demands rather than public need*” [90] (p. 4). Faber refers to the networks of powerful interest groups that undermine environmental progress as the “polluter-industrial complex”. These are think tanks, policy institutes, research centres, foundations, legal associations, political action committees, public relations firms etc., who are “*committed to discrediting the environmental movement and to dismantling state programs and policies that promote environmental justice, protect public health, and safeguard the earth*” [91] (p. 15). He describes how these groups, financed by corporate bodies, have dominated environmental debates in the US, appearing to express objective, factual, expert-based opinion, whilst working for particular economic interests.

Hence, in relation to the recycling examples given earlier, attempts to reduce waste have largely failed. For example, in the US where legislators have attempted to re-introduce returnable bottles, corporations such as Coca-Cola have spent millions of dollars to block the measures [92]. Furthermore, in another relevant example, attempts to highlight the link between toxic production methods and poor health have also been undermined. Corporate interests who are involved in the main cancer charities lobby to ensure that the focus is kept wholly on lifestyle factors and genes rather than the activities of the corporations that use toxic substances [93]. Hall alleges:

*A firm alliance between the established cancer institutions and the chemical, pharmaceutical and nuclear industries has formed the medical-industrial complex...What is stopping us [from getting serious about prevention] is the almost suffocating hold the medical-industrial complex retains over cancer policy, and the hugely powerful chemical industry’s interest in protecting its products*.

[93] (p. 62)

Therefore, with regard to breast cancer, there are few moves to ban the production of the relevant hazardous chemicals and pass legislation to protect the health of workers and communities.

In general, environmental reforms remain limited, allowed a marginal existence only insofar as they do not interfere with the priorities of the economic system.

Moreover, patriarchy is embedded in these processes of capitalism. Men, unrestricted by reproduction responsibilities have built capitalism along gender lines, contributing to gender inequality. Hence, women have often been drawn into labour under exploitative conditions. The environmental injustices emanating from capitalism are overlain with patriarchy, leading to the exploitation of women in their workplace and at home and a sexual division of labour, characterised by a hierarchy of activities in which “masculine” tasks are assigned high value and “feminine” ones, low value. This is further explored in eco-feminist literature which discusses how the presence of dualism in western culture contributes to the social structure of patriarchal society, e.g., [94]. Hence, combined capitalism and patriarchy facilitate gender-based environmental justice. This analysis indicates that the problem of environmental injustice on the basis of gender is systemic and, therefore, that it must be addressed through radical social change as discussed in the next section.

### 4.2. Solutions to Gender-Based Environmental Injustice

Gender mainstreaming was established as a major global strategy for the promotion of gender equality from the Fourth United Nations World Conference on Women 1995 [95]. The United Nations Economic and Social Council (*ECOSOC*) agreed conclusions 1997/2 defines gender mainstreaming as:

*“...the process of assessing the implications for women and men of any planned action, including legislation, policies or programmes, in all areas and at all levels. It is a strategy for making women’s as well as men’s concerns and experiences an integral dimension of the design, implementation, monitoring and evaluation of policies and programmes in all political, economic and societal spheres so that women and men benefit equally and inequality is not perpetuated. The ultimate goal is to achieve gender equality”*.

[95] (p. 9)

However, proposals to address environmental justice in relation to gender thus far appear somewhat vague. For example, the World Health Organisation states:

Issues of environmental and social justice and gender mainstreaming should be given more careful consideration by national and local policy-makers. A fairer distribution of environment and health resources should be an integral responsibility of actors in the environment, spatial planning and sustainable development sectors...Countermeasures to prevent and mitigate inequalities must take into account the driving forces behind such inequalities. Thus action must be taken at multiple levels to:

- uncouple the link between social determinants and environmental inequalities through targeted actions focusing on the most vulnerable and disadvantaged population groups;

*- stop and reverse environmental inequality trends by providing healthy environments for all*.

[23] (p. v)

Later, the same document recommends government action to reduce social inequalities in environmental risks through efforts to:

- improve daily living conditions
- tackle the inequitable distribution of power, money and resources
- *measure and understand the problem and assess the impact of action* [23] (p. 14).

Such approaches have the potential to draw women into environmental decision-making and address the factors that cause gender-based environmental injustice. However, research by Buckingham and Kulcur [7] found that these policies have been insufficient to ensure gender justice with regard to the environment. The WHO and UN recommendations imply some radical changes but they do not explicitly critique the economic and structural sources of the unhealthy and inequitable living conditions.

I consider that such policies are unlikely to be successful unless they address capitalism and patriarchy as the underlying causes of the poverty and inequality that result in women’s environmental injustice. If capitalist processes underlie and exacerbate environmental injustice, this would imply the need to transform or reject these processes.

Environmental justice can be achieved through collective and equal democratic control over the economy, where the needs of society and the environment are prioritised and labour and resources are redeployed to these ends. Wrenching humanity free of the strictures of capitalism, patriarchy and other modes of structural oppression will require that people be informed and willing to think independently and to share their knowledge and thinking. As Naomi Klein [96] has argued, capitalism, as a key factor undermining environmentalism, is best challenged by social movements identifying common cause. If feminist, environmental, anti-capitalist and other equalities groups could understand and articulate how their struggles intersect, their united strength could lever dramatic change. Hence, for the sake of humanity and the rest of nature, those of us working to achieve women’s emancipation and those striving for environmental justice, must establish mutual understanding and respect. The environmental justice literature can support this project by drawing on, and extending, feminist research on environmentalism. Similarly, environmental justice academics can benefit from more engagement with feminist literature. It is also important to combine this analysis with intersectional issues so as to capture the form and extent of environmental injustices as people experience them. I have not managed to do this here as I have endeavoured to look at a broad topic area, but more detailed analysis should certainly do this.

## 5. Conclusions

Environmental justice is a contested concept. It continues to expand and deepen but much remains to be said and stronger linkages can be formed with other justice issues. In particular, a gender dimension to environmental justice needs to be more thoroughly explored and articulated. Women experience distributional, procedural and substantive environmental injustice, lacking access to environmental resources; carrying an inequitable burden of environmental harm; and being constrained in terms of influence over environmental decision making. This is an attack on women’s psychological, physical, economic, and social wellbeing, sometimes ultimately resulting in death. The evidence presented here suggests that the underlying explanations for these environmental injustices are women’s lower income and status (relative to men). Both of these are perpetrated and perpetuated by capitalism entwined with patriarchy. The exploitation of women in their workplace and at home; the sexual division of labour; and the devaluing of women in society restricts their choices and undermines their ability to shape society according to their needs. This means that women are so that less able to avoid environmental problems. To help expose, so as to eventually dismantle, these processes, environmental research, policy-making and activism needs to link to their counterparts in the fields of equalities, social justice and public health. Such analysis and action will enhance our ability to physically survive, as well as to create a more beautiful and caring world so that“Yes, bread we fight for—but we fight for roses too”.
